# Structural, thermodynamic, and magnetic properties of SrFe_12_O_19_ hexaferrite modified by co-substitution of Cu and Gd

**DOI:** 10.1039/d3ra08878b

**Published:** 2024-03-01

**Authors:** Md. Roni Islam, M. K. R. Khan, Md. Sarowar Hossain, M. M. Rahman, M. Mahbubul Haque, M. Aliuzzaman, M. K. Alam, M. S. I. Sarker

**Affiliations:** a Department of Physics, Rajshahi University Rajshahi-6205 Bangladesh samiul-phy@ru.ac.bd; b Department of Physics, Faculty of Science and Technology (FST), American International University-Bangladesh (AIUB) Dhaka-1229 Bangladesh; c Materials Science Division, Atomic Energy Centre Dhaka-1000 Bangladesh; d Nuclear Power and Energy Division, Bangladesh Atomic Energy Commission Dhaka-1207 Bangladesh; e Department of Physics, Bangladesh University of Engineering & Technology Dhaka-1000 Bangladesh

## Abstract

A hard magnetic system of SrFe_12_O_19_ nanomaterial was modified according to the composition of Sr_0.95_Gd_0.05_Fe_12−*x*_Cu_*x*_O_19_ with *x* = 0.0, 0.30, and 0.60 using the sol–gel technique. The structures of the samples were evaluated using X-ray diffraction (XRD) along with Rietveld refinement, and an M-type hexaferrite with a hexagonal structure was confirmed with a trace amount of the α-Fe_2_O_3_ phase. In addition, transmission electron microscopy (TEM) analysis revealed polycrystalline nanoplates in all samples. Furthermore, the bond structures of the octahedral and tetrahedral sites along with the thermodynamic properties of these ferrites were extracted from the FTIR spectra at room temperature. The Debye temperature (*θ*_D_) decreased from 755.9 K to 749.3 K due to the co-substitution of Gd^3+^ at Sr^2+^ and Cu^2+^ at Fe^3+^. The magnetic hysteresis (*M*–*H*) measurements revealed that the coercivity decreased from 5.3 kOe to 1.5 kOe along with the highest magnetization saturation (*M*_s_) of 65.2 emu g^−1^ for the composition Sr_0.95_Gd_0.05_Fe_11.7_Cu_0.3_O_19_, which is suitable for industrial application. The effect of local crystalline anisotropy in magnetization was explored using the law of approach to saturation (LAS). Finally, thermo-magnetization was recorded in the range from 400 K to 5 K for cooling under zero field and in the presence of a 100 Oe field, and magnetic transitions were tracked due to the introduction of the foreign atoms of Gd and Cu into SrFe_12_O_19_.

## Abstract 2

A hard magnetic system of SrFe_12_O_19_ nanomaterial was modified according to the composition of Sr_0.95_Gd_0.05_Fe_12−*x*_Cu_*x*_O_19_ with *x* = 0.0, 0.30, and 0.60 using the sol–gel technique.

## Graphical abstract

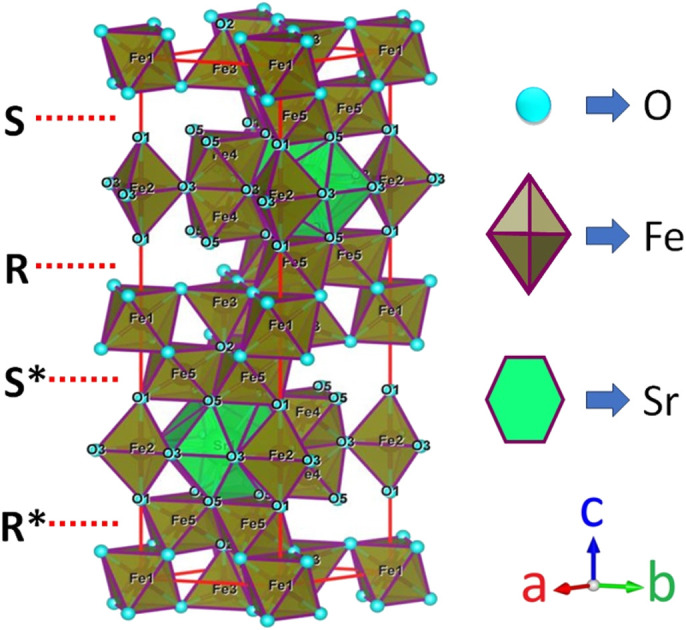

## 1. Introduction

**Fig. 1:**
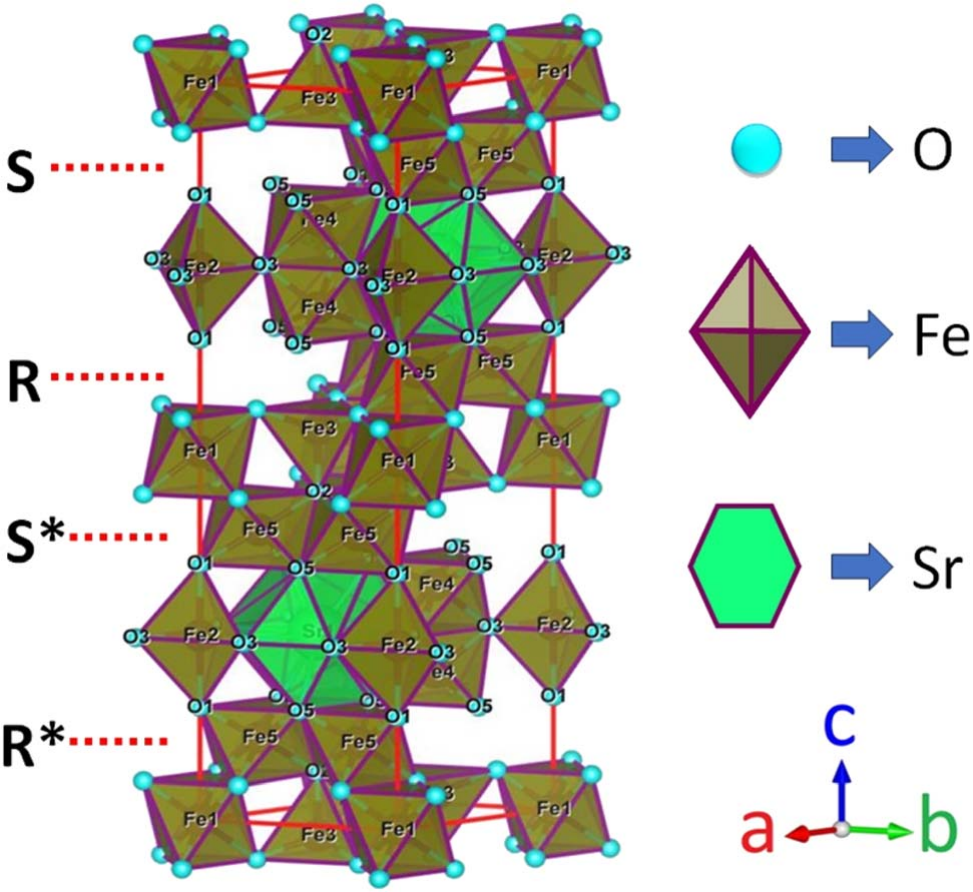
A diagram of the M-type hexaferrite structure for Fe^3+^ ions arranged in five different positions.

M-type Sr-hexaferrite (SrFe_12_O_19_), SFO, was first discovered in the Philips research laboratory^1^ and due to its hard magnetic properties and chemical stability, along with cost-effective production, it has attracted a lot of attention.^2^ As hard magnetic materials, there are numerous engineering uses for M-type hexagonal ferrites, MFe_12_O_19_ (where M = strontium (Sr), barium (Ba) and lead (Pb)), including magnetic recording media, microwave devices, and high-frequency applications.^3^ The hexaferrite structure can be divided into three basic block sequences, namely, spinel (Fe_6_O_8_)^2+^, hexagonally packed (SrFe_6_O_11_)^2−^–R block, and SRS*R* block, which are divided into the following types: M, Z, Y, W, X, and U.^4^ The strontium hexaferrite (SrFe_12_O_19_) crystallizes with a hexagonal magneto-plumbite structure and belongs to the space group *P*6_3_/*mmc*.^5^ The 24 Fe^3+^ atoms in the unit cell are spread among five different places in the hexagonal structure, which has two chemical units. Fig. 1 shows three octahedral symmetry sites (12k, 2a, and 4f2), one bipyramidal site (2b), and one tetrahedral symmetry site (4f1). The magnetic characteristics of M–hexaferrite depend on the orientation of the magnetic moment in the sub-lattices.^6^ A lot of research was carried out regarding its advantageous magnetic properties such as magnetic saturation, magnetic hardness, and Curie temperature. The magnetism of hexaferrite is strongly influenced by shape, magneto-crystalline anisotropy, and average crystallite size.^7^ Since a few decades ago, M-type hexaferrites have been used to replace rare earth (Nd, Gd, Ho, *etc.*) and d-block (Co, Ti, Ni, Cu, Mn, *etc.*) elements to enhance the magnetic and dielectric properties.^8^ The doping or substitution of foreign elements in the structure of M-type strontium hexaferrites enhances the magnetic behavior, absorption of microwaves, quality, ferromagnetic resonance frequency, and so on. Numerous investigations with a similar focus were carried out, and one of them found that bimetallic La–Co substitution was the best way to increase magneto-crystalline anisotropy without altering saturation magnetization M_s_.^9^ According to the Gorter model,^10^ the superexchange interactions *via* O^2^ anions couple sixteen ferric ion moments of magnetic attraction (12k, 2a, and 2b) parallel to the *c*-axis, resulting in ferrimagnetic ordering. The outcomes of first-principles calculations on magnetic structure have verified the concept. Andrzej Hilczer^6^ examined how doping with Sc affected the coercivity, remanence, and dielectric properties of SrM hexaferrites. Shakoor *et al.* added Bi–Cr to the strontium hexaferrites with interesting results and reported^11^ that according to the XRD data, the material contains a single magnetoplumbite phase, and the crystallite size ranges from 41 to 57 nm. The isolated disadvantage of La–Co and Bi–Cr-replaced M-type ferrites is the cost associated with the adding process, which uses pricey metals like La and Bi. Intense research is being done on these materials since it is still challenging to create low-cost ferrites with improved magnetic properties.^10,12^ M. Elansary *et al.*^13^ reported the effects of doping Gd^3+^, Sm^3+^, and transition elements (M = Ni, Zn, Mn, and Mg) on the structural, magnetic, and morphological properties of Sr_0.9_M_0.1_Fe_11.98_Sm_0.01_Gd_0.01_O_19_. The nanoparticles with the composition BaFe_12−3*x*_Gd_*x*_Sm_*x*_Y_*x*_O_19_ for *x* = 0, 0.01, 0.02 were synthesized by I. Lisser *et al.*^14^ using the sol–gel auto-combustion method. In addition, a single-phase hexaferrite of composition Sr_(1−*x*)_La_*x*_Gd_*y*_Sm_*z*_Fe_(12−(*z*+*y*))_O_19_ (*x* = 0.3, *y* = *z* = 0.01) was synthesized by the same method^15^ and the sizes of the particles were observed to vary from 53 nm to 46 nm. A ternary dopant, Gd–Ho–Sm, was implemented to synthesize a single phase of M-type Sr hexaferrite of 49 nm particles in a cost-effective way.^16^ It has been reported that Gd^3+^ and Ho^3+^ ions have strong preferences towards the 12k site, whereas the Sm^3+^ ions prefer to occupy the 2A site of the lattice. Another sample of Al-SFO exhibited good catalytic activity compared to the parent compound due to the presence of Al^3+^ ions in the octahedral sites, and these sites are exposed to the surface of the strontium hexaferrite catalyst.^17^ Moreover, catalytic activity could be induced in the hard magnetic strontium hexaferrite sample by replacing the small fraction of Fe with Cu.^18,19^ Therefore, SrFe_12_O_19_ powders of various forms and sizes have been made using a variety of procedures, including the sol–gel, hydrothermal, co-precipitation of chemicals, solid-state reaction, and micro-emulsion approaches, among others. One of the practical ways to crystallize the hexaferrite phase at a comparatively lower annealing temperature is to create SrFe_12_O_19_ nanoparticles (NPs) using the sol–gel process.^12^ Sol–gel technology is extensively used as a great way to create nanospinels because of its advantages of low processing costs, energy efficiency, high production rates, and the production of fine homogeneous powder.^11^ Incorporating metallic ions like Gd^3+^ and Cu^2+^ into the hexaferrite has produced some interesting outcomes. Cu^2+^ prefers to occupy the octahedral 4f2 position, which has a down-spin state and contributes adversely to the overall saturation magnetization.^20^ However, the addition of any foreign elements to Sr hexaferrites not only improves their physical properties, but different types of anisotropy may also develop inside, which limits their applications.

The present work includes an in-depth investigation of the structural, thermodynamic, magneto-anisotropic, and thermo-magnetic properties of SrFe_12_O_19_ nanoparticles modified by the co-substitution of a rare earth element, Gd^3+^, at Sr^2+^ and a transition element, Cu^2+^, at Fe^3+^.

## 2. Experimental details

### 2.1. Sample preparation

The M-type hexaferrite, SrFe_12_O_19_ (parent sample), was modified by the substitution of Gd at Sr, and Cu at Fe, and the compositions of Sr_0.95_Gd_0.05_Fe_12−*x*_Cu_*x*_O_19_ for *x* = 0.30, 0.60 were synthesized. The raw materials of analytical grade strontium nitrate [Sr(NO_3_)_2_] and gadolinium nitrate [Gd(NO_3_)_2_·5H_2_O] were obtained from LOBA Chemise, ferric nitrate [Fe(NO_3_)_2_·9H_2_O] from E. Merck, and copper nitrate [Cu(NO_3_)_2_·5H_2_O], 99.9% from ALDRICH. In addition, citric acid [C_6_H_8_O_7_·H_2_O] of 99% purity, from E. Merck and HCl were used as chelating agents. The stoichiometric amounts of 0.190 g (0.03 M) Sr(NO_3_)_2_, 4.3632 g (0.03 M) Fe(NO_3_)_2_·9H_2_O and 0.190 g (0.03 M) C_6_H_8_O_7_·H_2_O (1 : 12 : 1 for Sr, Fe and citrate) were dissolved at a room temperature in 30 ml distilled water (98%) for 2 h to manufacture undoped SrFe_12_O_19_. After that, the solution was evaporated using a water bath to speed up the gelation process. The dehydration process was performed over 6 hours, and after that, a fine dried gel was produced over 24 hours in ovens set at 400 K. Through intermediate grinding, the dried gel of the components was finely mixed with oxides. Both SrFe_12_O_19_ and doped powder samples were obtained after calcination at 1023 K in a furnace. In this investigation, the synthesized SrFe_12_O_19_ was identified as SFO. The other two compositions of Sr_0.95_Gd_0.05_Fe_11.4_Cu_0.6_O_19_, and Sr_0.95_Gd_0.05_Fe_11.7_Cu_0.3_O_19_ are presented herein as SGFCO-1 and SGFCO-2, respectively.

### 2.2. Characterization

The thermal stability of the as-prepared parent sample, SFO, was confirmed and differential thermal analysis (DTA) and thermogravimetric (TG) measurements were performed in a PerkinElmer STA-8000 °C system at a heating rate of 10 K min^−1^ under a nitrogen (N_2_) atmosphere. The structures of SFO, SGFCO-1 and SGFCO-2 samples were evaluated using an X-ray diffractometer (PW3040) with Cu-K_α_ radiation (*λ* = 1.5405 Å) and the diffraction patterns were recorded in the range of 20° ≤ 2*θ* ≤ 70° at room temperature (RT = 300 K). The microstructures of the studied samples, along with selected area diffraction patterns (SAED), were determined using a Tecnai G2 30ST transmission electron microscope (TEM). Elemental studies of the synthesized samples were conducted using an energy-dispersive X-ray spectrometer (EDS) attached to the TEM and the measurements were performed for 5 different locations of the overall microstructure. Moreover, the bond structure and thermodynamic properties of all samples were evaluated by Fourier transform infrared spectroscopy (FTIR) on a Nicolet NEXUS 470 FTIR Spectrometer in the range of 350–3700 cm^−1^ at RT. Finally, the magnetic hysteresis (*M*–*H* loop) at RT and magnetization as a function of temperature (*M*–*T*) ranging from 10 K to 400 K were determined for all studied samples using a Quantum Design PPMS. For the *M*–*H* loop measurement, the highest limit of the magnetic field (*H*) was ±20 kOe and for the *M*–*T* measurement, the rate of cooling (d*T*/d*t*) was 2 K s^−1^.

## 3. Results and discussion

### 3.1. Thermal stability

**Fig. 2:**
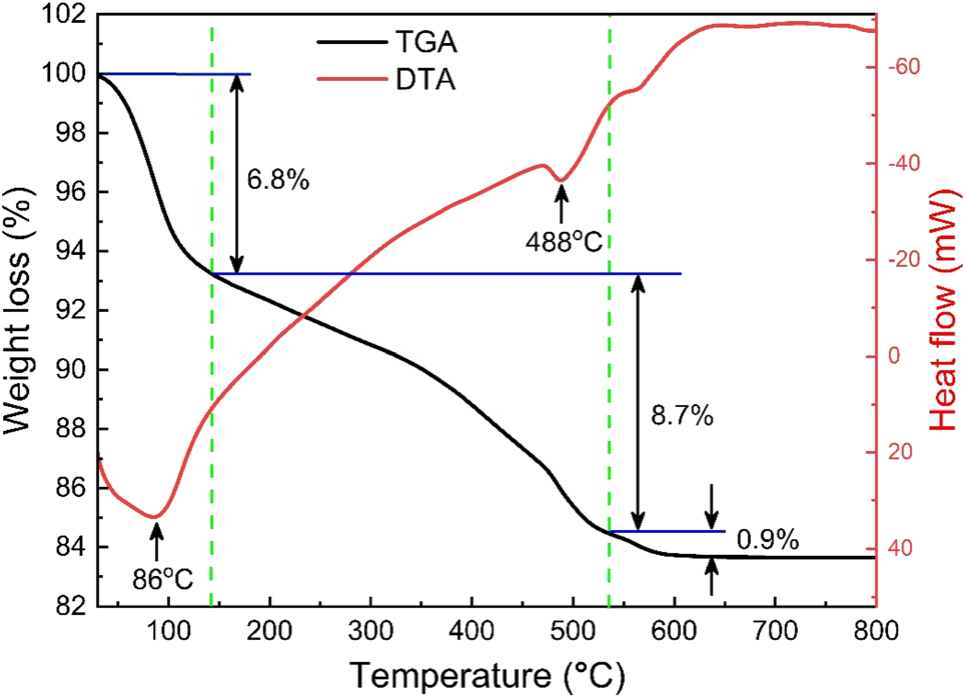
TG and DTA curves of the as-synthesized SrFe_12_O_19_.

The thermogravimetric (TG) and differential thermal analysis (DTA) of the as-prepared SrFe_12_O_19_ sample ensured the formation and phase stability of the synthesized sample. Fig. 2 displays the TG and DTA curves between RT and 1000 K for the parent SFO sample and the decomposition of the ingredients was observed due to a thermally activated chemical reaction. However, this decomposition followed several steps at elevated temperatures and the first step of 6.8% weight loss was observed between RT and 415 K, which was attributed to a trace quantity of chelating compound with ammonia. Upon further heating to 810 K, the evaporation of the remaining solvent and the crystallization process were ascribed to a considerable weight loss of 8.7% as a second step. Beyond that, a final step of 0.9% weight loss was observed up to 1000 K and 83.6% residue remained after thermal analysis in the overall temperature range. Two endothermic peaks at 360 K and 760 K are likely due to water loss and Sr^2+^ and Fe^2+^ decomposition, respectively. This demonstrates the thermal stability of the synthesized SFO hexaferrite NPs.

### 3.2. Structural characterization

**Fig. 3:**
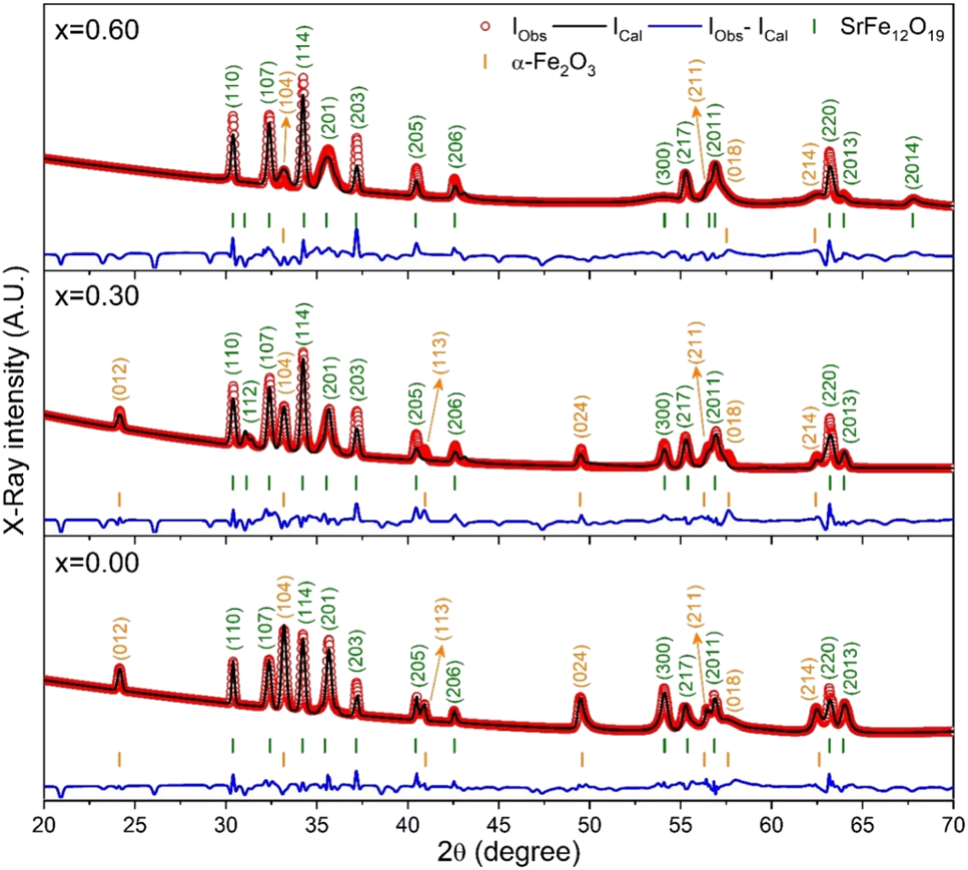
XRD patterns (*I*_Obs_) recorded for the SrFe_12_O_19_ and Sr_0.95_Gd _0.05_Fe_12−*x*_Cu_*x*_O_19_ compositions (*x* = 0.30 and 0.60) along with the calculated patterns (*I*_Cal_), differences between the observed patterns and calculated patterns (*I*_Obs_ − *I*_Cal_) and peak positions (vertical bar) obtained by Rietveld refinement.

The X-ray diffraction (XRD) patterns for the synthesized SrFe_12_O_19_, Sr_0.95_Gd_0.05_Fe_11.4_Cu_0.6_O_19_, and Sr_0.95_Gd_0.05_Fe_11.7_Cu_0.3_O_19_ were recorded at RT and the patterns are shown in Fig. 3. The arrangements of diffraction patterns ensure the formation of the crystalline phase in all the samples. Therefore, the crystallographic planes and structural parameters along with the phase fractions were extracted from the analysis of XRD data using the Rietveld refinement method by FullProf Suite software.^21,22^Fig. 3 shows the fitting of diffraction patterns, where the experimental data (*I*_Obs_) are depicted by the red circles, the black lines represent calculated intensities (*I*_Cal_), and the blue lines represent (*I*_Obs_ − *I*_Cal_). The Bragg positions are displayed by the green and orange vertical lines. Here, the quality-based fitting of XRD data has been determined by *χ*^2^, which is between 2.35 and 5.54. The other fitting factors are *R*_p_ (residual of least squares refinement) and *R*_wp_ (weighted profile factor), which are also limited. From the analysis of the XRD peak matching, a major part of the patterns matches the *P*6_3_/*mmc* space group, while few of them fit the *R*3̄*c* space group, which indicated the presence of two identical phases inside the synthesized samples. However, the respective arrangements of the odd/even lattice peaks of (110), (112), (107), (114), (201), (203), (205), (206), (300), (217), (2011), (220), and (2013) reflect the M-type hexaferrites with hexagonal structure from the *P*6_3_/*mmc* space group (JCPDS Card No. 79-1411).^12^ The other phase includes the arrangements of the odd/even lattice peaks of (012), (104), (113), (024), (211), (018), (224), which are reflected from the α-Fe_2_O_3_ of the rhombohedral structure from the *R*3̄*c* space group (JCPDS Card No. 33-0664).^23^ However, from close observation of the patterns, the elimination of the impurity phase (α-Fe_2_O_3_) is due to the co-substitution of Gd and Cu in the parent SFO sample. Apart from this, the structural parameters have been included in Table 1. The obtained experimental values of the lattice constants were *a* = *b* = 5.8761 Å and *c* = 23.0239 Å for the parent SFO sample, and the reported values for the same composition were 5.8751 Å and 23.0395 Å, respectively,^24^ where the synthesis conditions are responsible for the differences. However, the lattice parameters for the major phase increased due to the substitution of Gd^3+^ at Sr^2+^ and Cu^2+^ at the place of Fe^3+^, but the variation is very marginal in *a*, *b* and *c*. The ionic radius of Gd^3+^ (93.8 pm) is smaller than that of Sr^2+^ (118 pm) for a coordination number of six, according to the database of ionic radii provided by R. D. Shannon.^25^ Therefore, the lattice parameters (*a*, *b* and *c*) are supposed to decrease in SGFCO due to the substitution of Gd^3+^ at Sr^2+^. On the other hand, the ionic radius for Cu^2+^ (73 pm) is larger than Fe^3+^ (64.5 pm) for a coordination number of six.^25^ Consequently, the lattice parameters (*a*, *b* and *c*) are supposed to increase in SGFCO due to the substitution of Cu^2+^ at Fe^3+^. Since the substitution of Cu^2+^ is greater than Gd^3+^ in the parent sample, the lattice parameters increased. However, the increase is marginal even though more Cu was substituted in the SGFCO sample. The overall variations increased the unit cell volume (*V*) in the SGFCO-1 and SGFCO-2 samples. The percentage of the existing phases, *W*_P_ (%) was determined from equation:^22^1
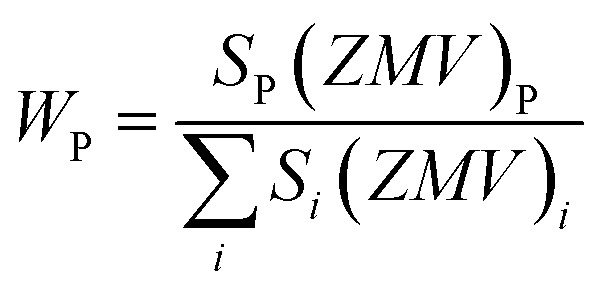
where the parameters of the unit cell volume (*V*), the formula unit of the unit cell (*Z*), formula unit mass (*M*) and scale factor (*S*) were determined from the Rietveld refinement. Table 1 presents the number of phases (%) in the studied samples and the impurity phase, α-Fe_2_O_3_, decreased from 27.2% to 17.9% for the replacement of Sr^2+^ by Gd^3+^, and Fe^3+^ by Cu^2+^ with an amount of 5% in both cases. Here, the synthesized powder samples were calcined at 1023 K (750 °C) and the presence of a secondary phase of hematite (α-Fe_2_O_3_) indicates an incomplete reaction. The reported minimum energy required to transform the oxide compounds SrO and Fe_2_O_3_, to produce the SrFe_12_O_19_ phase was in the temperature range of 711–878 °C.^26^ In another article by H. M. Shashanka *et al.*, the single-phase Sr-hexaferrite was produced with a calcination temperature of 1200 °C for 2 h.^27^ In an earlier report by M. A. Urbano Peña *et al.*,^28^ a secondary phase of α-Fe_2_O_3_ was observed in SrFe_12_O_19_ samples calcinated at 800 °C and the samples were synthesized by the Pechini method. Therefore, the presence of a secondary phase (α-Fe_2_O_3_) in pure SrFe_12_O_19_ depends not only on the calcination temperature but also on the synthesis conditions and the presence of catalysts in the reaction environment. Moreover, the replacement of Cu at Fe in SrFe_12_O_19_ led to a decrease in the phase formation temperature as the melting point of Cu is 1312 °C, whereas the melting point for Fe is 1535 °C. On the other hand, the melting point of Gd is 1084 °C, whereas the melting point of Sr is 768.8 °C. Therefore, the replacement of Gd at Sr led to an increase in the phase formation temperature. As a result, the mutual effect of the co-substitution of Cu and Gd created a complex situation during the phase formation of the pure hexaferrite phase of SrFe_12_O_19_. From the viewpoint of Cu substitution only, the hexaferrite phase of SrFe_12_O_19_ achieved a more favourable environment from the calcination temperature of 750 °C. Therefore, a low rate of secondary phase was observed in the SGFCO-1 sample and a further decrease in the amount (%) was observed due to the replacement of more Fe by Cu atoms (SGFCO-2). Finally, sample SGFCO-2 with a smaller amount of the α-Fe_2_O_3_ phase (17.9%) showed the highest value of the lattice parameter as compared to the other two samples since a smaller amount of Fe^3+^ departed from the parent phase to form the secondary phase of the α-Fe_2_O_3_ phase.

**Table 1:** Structural parameters of SrFe_12_O_19_ (SFO), Sr_0.95_Gd_0.05_Fe_11.7_Cu_0.3_O_19_ (SGFCO-1), and Sr_0.95_Gd_0.05_Fe_11.4_Cu_0.6_O_19_ (SGFCO-2) showing crystallite size (*D*_114_) and lattice strain (*ε*), phase percentages (wt%), lattice parameters (*a*, *b* and *c*), and unit cell volume (*V*) along with parameters for Goodness of fit (*χ*^2^), residuals for unweighted pattern (*R*_p_) and weighted pattern (*R*_wp_)

Sample	*D*_114_ (nm)	*ε*	Crystal structure	Phase percentages (wt%)	Lattice parameters (Å)	*V* (Å^3^)	*R* Factor (%)	*χ* ^2^
SFO	42.5	0.0029	SrFe_12_O_19_	72.8 ± 0.9	*a* = *b* = 5.8761 (3)	688.5 (1)	*R*_p_ = 8.7	2.35
			*P*6_3_/*mmc* (hexagonal)		*c* = 23.0239 (3)		*R*_wp_ = 7.2	
			α-Fe_2_O_3_	27.2 ± 0.5	*a* = *b* = 5.0316 (2)	301.1 (1)	*R*_p_ = 6.8	
			*R*3̄*c* (Rhombohedral)		*c* = 13.7285 (2)		*R*_wp_ = 5.9	
SGFCO-1	39.2	0.0031	Sr_0.95_Gd_0.05_Fe_11.7_Cu_0.3_O_19_	78.6 ± 1.0	*a* = *b* = 5.8801 (6)	689.5 (1)	*R*_p_ = 9.8	3.98
			*P*6_3_/*mmc* (hexagonal)		*c* = 23.0269 (3)		*R*_wp_ = 9.1	
			α-Fe_2_O_3_	21.4 ± 0.3	*a* = *b* = 5.0372 (6)	301.9 (1)	*R*_p_ = 8.9	
			*R*3̄*c* (rhombohedral)		*c* = 13.7397 (3)		*R*_wp_ = 8.3	
SGFCO-2	43.0	0.0029	Sr_0.95_Gd_0.05_Fe_11.4_Cu_0.6_O_19_	82.1 ± 1.5	*a* = *b* = 5.8809 (7)	689.9 (2)	*R*_p_ = 10.3	5.54
			*P*6_3_/*mmc* (hexagonal)		*c* = 23.0347 (4)		*R*_wp_ = 9.6	
			α-Fe_2_O_3_	17.9 ± 0.6	*a* = *b* = 5.0431 (9)	302.6 (2)	*R*_p_ = 9.2	
			*R*3̄*c* (rhombohedral)		*c* = 13.7368 (8)		*R*_wp_ = 7.1	

This discrepancy in the phase amounts will affect the other physical properties, including the magnetic properties, of the synthesized samples. Moreover, other structural factors like crystallite sizes and porosities also play a vital role in the enhancement of the ferromagnetic behaviour of the ferrite samples.^29,30^ The sizes of crystallites (*d*_114_) in all studied samples were estimated from the diffraction peak at (114), which represents the major phase (hexagonal) and calculation was performed by the Debye–Scherrer formula^31^ as expressed by the following equation:2
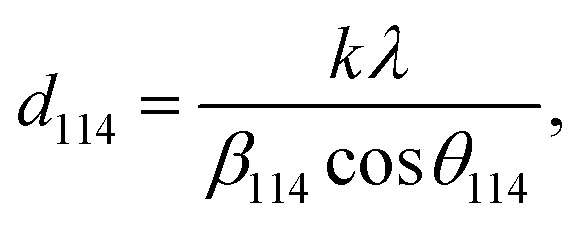
where *β*_114_ is the FWHM determined by the Gaussian fitting of the peak (114) at the Bragg position of *θ*_114_, *λ* = 1.5418 Å (wavelength of Cu-*k*_α_ radiation) and *k* = 0.9 (a dimensionless constant). The micro-strain (*ε*) of these crystallites was calculated using the following equation:^31^3
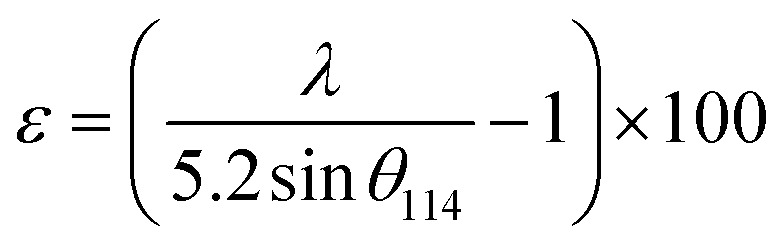


The obtained values of *d*_114_ and *ε* for all studied samples are included in Table 1 and marginal variations in the crystallite size of the major phase were observed.

### 3.3. Morphological analysis

**Fig. 4:**
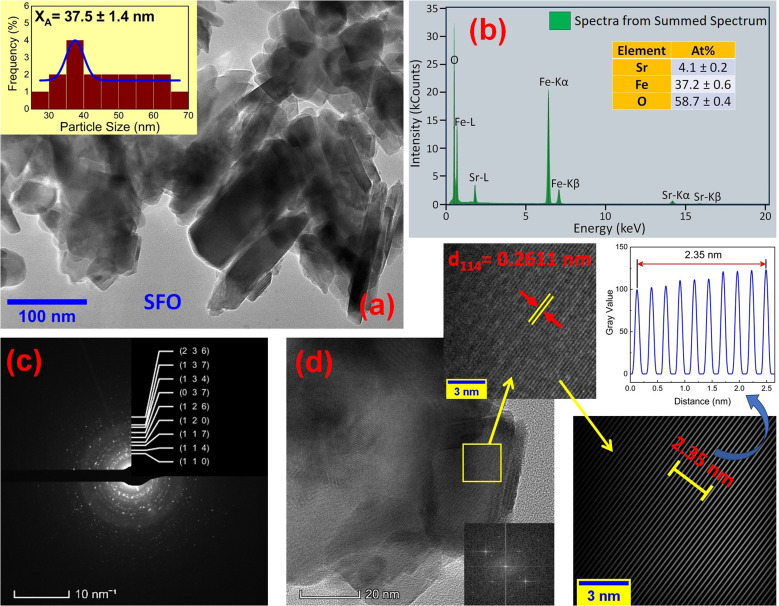
Microstructures obtained from transmission electron microscopy (TEM) showing (a) grain morphologies, (b) EDS spectra, (c) ring-type SAED patterns and (d) fast Fourier transform (FFT) patterns that demonstrate the poly-crystalline structure of the SrFe_12_O_19_ sample.

**Fig. 5:**
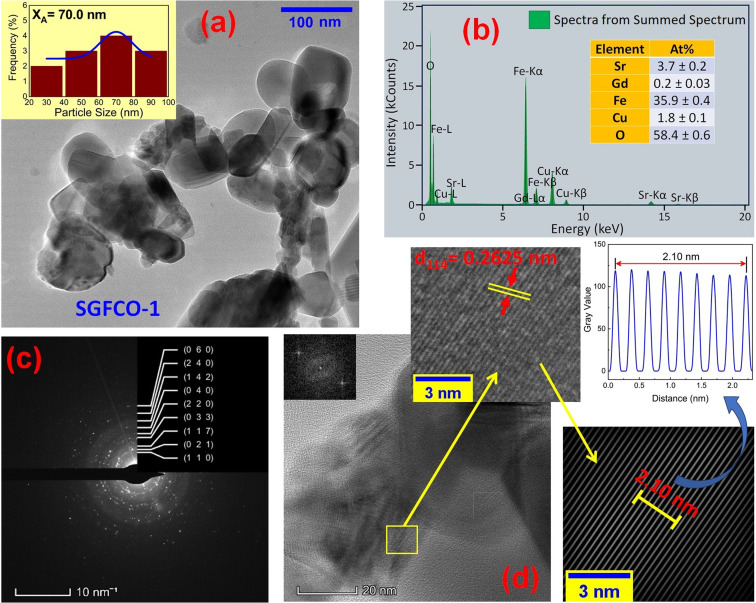
Microstructure obtained from transmission electron microscopy (TEM) showing (a) grain morphologies, (b) EDS spectra, (c) ring-type SAED patterns and (d) Fast Fourier transform (FFT) patterns that demonstrate the poly-crystalline structure of the Sr_0.95_Gd_0.05_Fe_11.7_Cu_0.3_O_19_ sample.

**Fig. 6:**
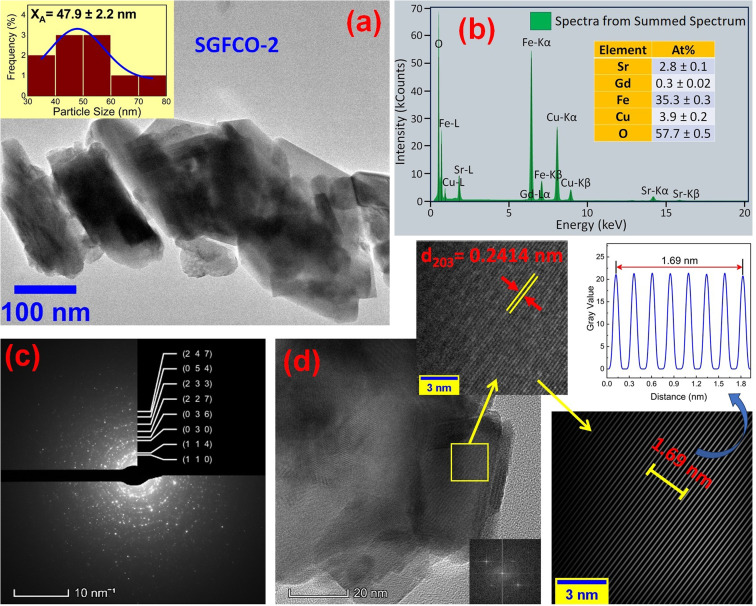
Microstructure obtained from transmission electron microscopy (TEM) showing (a) grain morphologies, (b) EDS spectra, (c) ring-type SAED patterns and (d) Fast Fourier Transform (FFT) patterns that demonstrate the poly-crystalline structure of the Sr_0.95_Gd_0.05_Fe_11.4_Cu_0.6_O_19_ sample.

The microstructures along with grain size distribution for SFO, SGFCO-1 and SGFCO-2 samples were observed using transmission electron microscopy (TEM) and Fig. 4(a), 5(a), and 6(a) display the surface morphologies of the studied samples. Here, the sizes of different grains have been scaled by the calibration of the line profile in the ImageJ 1.50i software and the fitting of the size distributions by the Lorentz function gives the average grain sizes (*X*_A_). The insets of Fig. 4(a), 5(a) and 6(a) represent the fitting of the size distributions of nanoplates that range from 20 nm to 100 nm, depending on the studied compositions. However, the overlapping of the platelets indicates the presence of axial magnetic interaction. The percentage of intergrain porosities (%) was calculated from *P*_i_ = [1 − (*ρ*_b_)/(*ρ*_x_)] × 100, where *ρ*_x_ is the theoretical density and *ρ*_*b*_ is the bulk density. Here, the value of *ρ*_x_ is connected to the molecular mass (*A*) and unit cell volume (*V*) by 
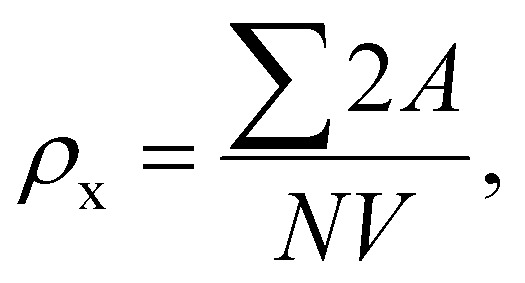
 where *N* is Avogadro's number. In addition, the value of *ρ*_b_ was measured from the bulk properties of the samples as *ρ*_b_ = mass/volume. Therefore, the estimated values of *P*_i_ are included in Table 2 and the lowest value of *P*_i_ was observed for the sample SGFCO-2 (*P*_i_ = 13.6%) along with the grains of 47.9 nm size. The compositions of the synthesized samples were determined from the EDS spectra as displayed in Fig. 4(b)–6(b) and Cu–Gd-substituted Sr hexaferrite compositions were identified from these results. The ring-shaped selected area diffraction patterns (SAED) as depicted in Fig. 4(c)–6(c) confirmed the formation of polycrystalline hexaferrites. These SAED patterns were indexed using CrysTBox Server software in which calibration was performed with the JCPDS Card No. 79-1411 (ref. 12) of the standard SFO sample. In parallel, the HR-TEM data represent the crystallinity of the samples. Therefore, the values of interplanar spacing (*d*), along with the crystal planes, were determined by the ImageJ 1.50i software using the FFT and inverse FFT methods of calculation. From Fig. 4–6, the values of *d* were observed as 0.2611 and 0.2625, corresponding to the [114] crystal planes of SFO and SGFCO-1, respectively while the value of *d* is 0.2414, corresponding to the [203] plane for SGFCO-2 samples.

**Table 2:** The estimated values of average grain size (*X*_A_), internal porosity (*P*_i_) (%), and therefore, the variation of force constants of the ions existing in tetrahedral and octahedral sites with FWHM of peaks at vibrational bands of octahedral (*W*_O_) and tetrahedral (*W*_T_) for SFO, SGFCO-1, and SGFCO-2 samples

Sample	*P*_i_ (%)	*X*_A_ (nm)	*k*_O_ (N m^−1^)	*k*_T_ (N m^−1^)	*k* ^Fe–O^_O_ (N m^−1^)	*k* ^Fe–O^_T_ (N m^−1^)	*W*_O_	*W*_T_	*L*_O_ (Å)	*L*_T_ (Å)	*L* ^Fe–O^_O_ (Å)	*L* ^Fe–O^_T_ (Å)
SFO	15.1	37.5	2288.4	2680.2	53.8	95.8	65.5	135.0	0.1951	0.1851	0.6809	0.5619
SGFCO-1	19.1	70	2235.5	2645.2	52.6	95.2	67.8	145.1	0.1966	0.1859	0.6862	0.5630
SGFCO-2	13.6	47.9	2212.3	2595.8	51.8	94.9	62.5	144.6	0.1973	0.1871	0.6895	0.5636

### 3.4. Elastic properties and thermal behaviour

**Fig. 7:**
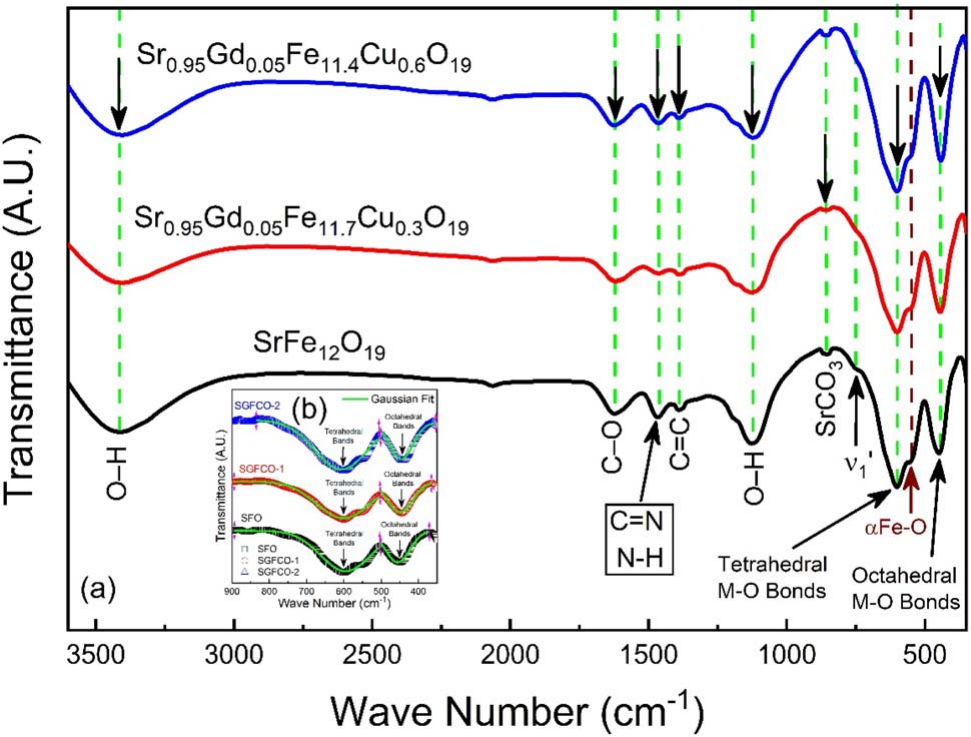
(a) Fourier-transform infrared spectroscopy for SrFe_12_O_19_, Sr_0.95_Gd_0.05_Fe_11.4_Cu_0.6_O_19_, and Sr_0.95_Gd_0.05_Fe_11.7_Cu_0.3_O_19_ samples scanned from 350 cm^−1^ to 3600 cm^−1^. (b) The inset shows the comparison between the peak width of the samples.

The elastic and thermodynamical properties of the SrFe_12_O_19_, Sr_0.95_Gd_0.05_Fe_11.4_Cu_0.6_O_19_, and Sr_0.95_Gd_0.05_Fe_11.7_Cu_0.3_O_19_ were determined from FTIR spectra obtained at RT. Fig. 7 shows the FTIR spectra obtained for the studied samples in the wavenumber range 350–3600 cm^−1^. Here, the absorption peaks at nearly 600 cm^−1^ and 447 cm^−1^ represent the main characteristic features of the synthesized samples, which are denoted by *ν*_A_ and *ν*_B_, respectively. The bands at around 600 cm^−1^ and 447 cm^−1^ originated due to oxygen motion at the tetrahedral (A-site) and octahedral (B-site) sites, respectively for the studied ferrites.^32^ The small band 
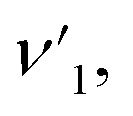
 near 754 cm^−1^, signifies the vibration of metal ions in the crystal lattice.^33^ In the synthesized sample, the broad bands at around 1120 cm^−1^ and 3415 cm^−1^ are attributed to the stretching vibrations of the O–H group of citric acid and molecular water.^12^ The band at 856 cm^−1^ is attributed to SrCO_3_. The band at 1634 cm^−1^ is assigned to the stretching vibrational band of the C=O group of CA.^34^ The band at around 1467 cm^−1^ corresponds to the vibrational modes of nitrate stretching.^35,36^ The C=C bond at 1388 cm^−1^ was observed due to the presence of CO_2_ during the heat treatment process.^37^ The overall bands around 400–600 cm^−1^ ensured the formation of the hexaferrite phase^38^ in all studied samples. However, a small vibrational band at 550 cm^−1^ is an indicator of the existing α-Fe_2_O_3_ phase,^12^ concomitant with the XRD data. The bond structure and force constants of the studied samples were extensively analysed from close observation of the absorption peaks *ν*_A_ and *ν*_B_. The widths of these peaks were compared by the Gaussian fitting (Fig. 7(b)). The widths of the peaks at the tetragonal and octahedral sites were denoted by *W*_T_ and *W*_O_, respectively, and the values are included in Table 2. *W*_T_ increased for both SGFCO-1 and SGFCO-2 samples, which implies that M–O bonds at the tetrahedral site are highly affected due to substitution of Gd^3+^ at Sr^2+^ and Cu^2+^ at Fe^3+^. There was no peak shoulder at *ν*_B_, confirming the presence of Fe^2+^ from the octahedral site.^39^ The slight shifting of *ν*_A_ and *ν*_B_ to the lower wavenumber indicates the perturbation in the Fe^2+^–O^2−^ bond that occurred for Gd^3+^ and Cu^2+^ substitution.^40^ The general equation for the force constant (*k*) of the metal–oxygen bond can be expressed by the following equation:^41^4
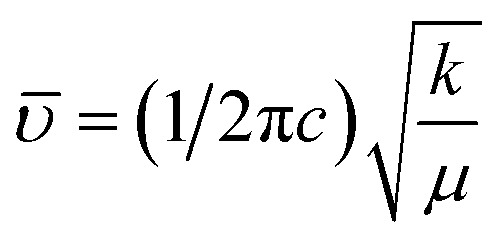
where *ῡ* is the wave number, *c* is the velocity of light, and *μ* is the effective mass. Eqn (4) has been used to measure the force constant of Fe–O bond at octahedral and tetrahedral sites and the effective mass for the bond is 
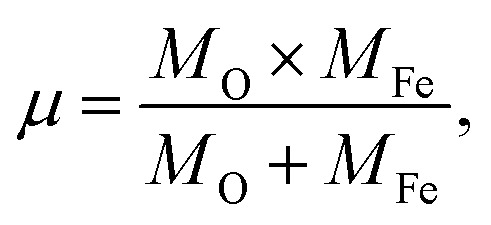
 where, *M*_O_ and *M*_Fe_ are the masses of O and Fe, respectively. The force constant at the octahedral site and tetrahedral site have been identified as *k*^Fe–O^_O_ and *k*^Fe–O^_T_, respectively and the values are included in Table 2. In addition, the bond length (*L*) of Fe–O was determined from the formula 
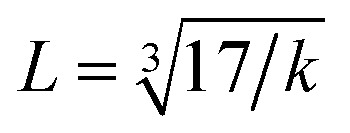
 for sites A and B and are included in Table 2 as denoted by *L*_A_ and *L*_B_, respectively.^41^ It was observed that *k*^Fe–O^_O_ and *k*^Fe–O^_T_ decreased for the co-substitution of Gd^3+^ and Cu^2+^ in SFO, which is reflected in the Fe–O bond length. Therefore, the overall force constants of M–O bonds at the octahedral site (*k*_O_) and tetrahedral site (*k*_T_) were determined from the following formulas:^42,43^

5 *k*_T_ = 7.62*M*_T_*ν*_A_^2^ × 10^−7^ (N m^−1^)

6 *k*_O_ = 10.62*M*_0_*ν*_B_^2^ × 10^−7^ (N m^−1^)

where *M*_T_ and *M*_O_ are the masses of the molecules at the tetrahedral and octahedral sites, respectively. The average cation–anion bond lengths in both sites were also estimated using the same formula, 
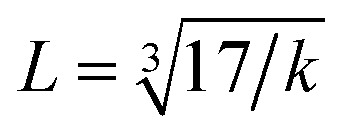
. The average force constant (*k*_av_) was used to estimate the elastic constants in this case. From the lattice constant (*a*) and *k*_av_, the stiffness constant (*C*_11_ = longitudinal modulus) was computed as *C*_11_ = *k*_av_/*a*.^44^ For the pore fraction, Poisson's ratio (*σ*) of the samples was calculated using the relation, *σ* = 0.324 × (1 − 1.043*f*).^45,46^ The values of *σ* exhibit a consistent divergence between 0.26 and 0.28 based on the compositions (Table 3) and the values fall within the range of −1 to 0.5, which is matched with the theory of isotropic elasticity. In addition, the stiffness constant *C*_12_ was calculated from *σ* and *C*_11_ using the following equation:7
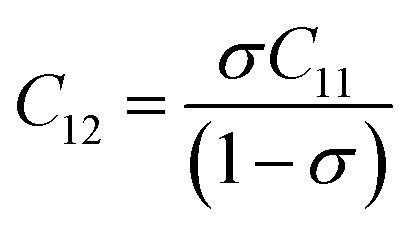


**Table 3:** Elastic properties of SFO, SGFCO-1, and SGFCO-2 showing Poisson's ratio (*σ*), Zener anisotropy (*Z*_A_), Debye temperature (*θ*_D_), Young's modulus (*E*), rigidity modulus (*G*), bulk modulus (*K*), elastic wave velocities for longitudinal (*v*_L_), transverse (*v*_T_) and mean velocity (*v*_m_)

Samples	*σ*	*Z*_A_	*v*_L_ (×10^3^) (m s^−1^)	*θ*_D_ (K)
SFO	0.27	0.57	2.81	755.9
SGFCO-1	0.26	0.59	2.79	751.6
SGFCO-2	0.28	0.56	2.76	749.3

The acquired values of *C*_12_ are positive and show the stability of the synthesized Gd-doped SFO hexaferrite. They range from 15.44 GPa to 15.88 GPa, depending on the compositions. The values of longitudinal elastic wave velocity (*V*_L_) were determined using the following equation:^46^8
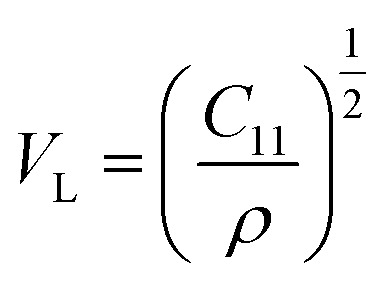
where *ρ* is the XRD density, as evaluated earlier. The change in *V*_L_ with Gd^3+^ replacement is presented in Table 3 and all the velocities are higher for the SGFCO-1 sample. In addition, the Debye temperature (*θ*_D_) is characteristic of a particular material that allows homogeneous isotropic massless phonons to dominate the thermal behavior of solids and it is the temperature at which phonons can have their highest frequency. The values of *θ*_D_ for the studied samples have been evaluated from the relation:^47,48^9
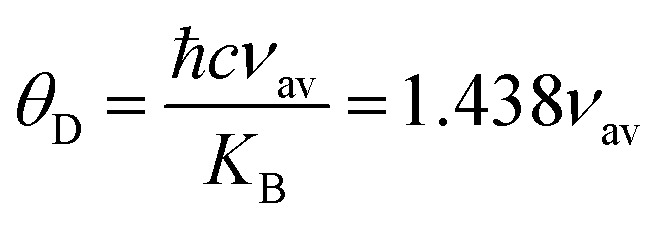
where ℏ is Planck's constant, *K*_B_ is Boltzmann's constant, *c* is the velocity of light, and *ν*_av_ is the average value of wavenumbers. The value of *θ*_D_ for SFO sample is 755.9 K which decreases with the increase in Cu^2+^ substitution. Table 3 represents the decrease in *θ*_D_ and longitudinal elastic wave velocity (*V*_L_) due to Gd^3+^ and Cu^2+^ substitution. Here, the decrease in *θ*_D_ indicates that the lattice vibrations held up for Gd^3+^ and Cu^2+^ substitution. The decrease in *θ*_D_ may be associated with the increase in the conduction electron density *N*_n_ (n-type). Hence, the density of conduction holes *N*_p_ (p-type) decreases.^49^ On the contrary, Anderson's formula depicts the linear increase in *θ*_D_ with *V*_m_.^46^ However, the synthesized SGFCO-1 ferrite sample is mostly porous, and anomalies were observed.

### 3.5. Magnetic hysteresis

**Fig. 8:**
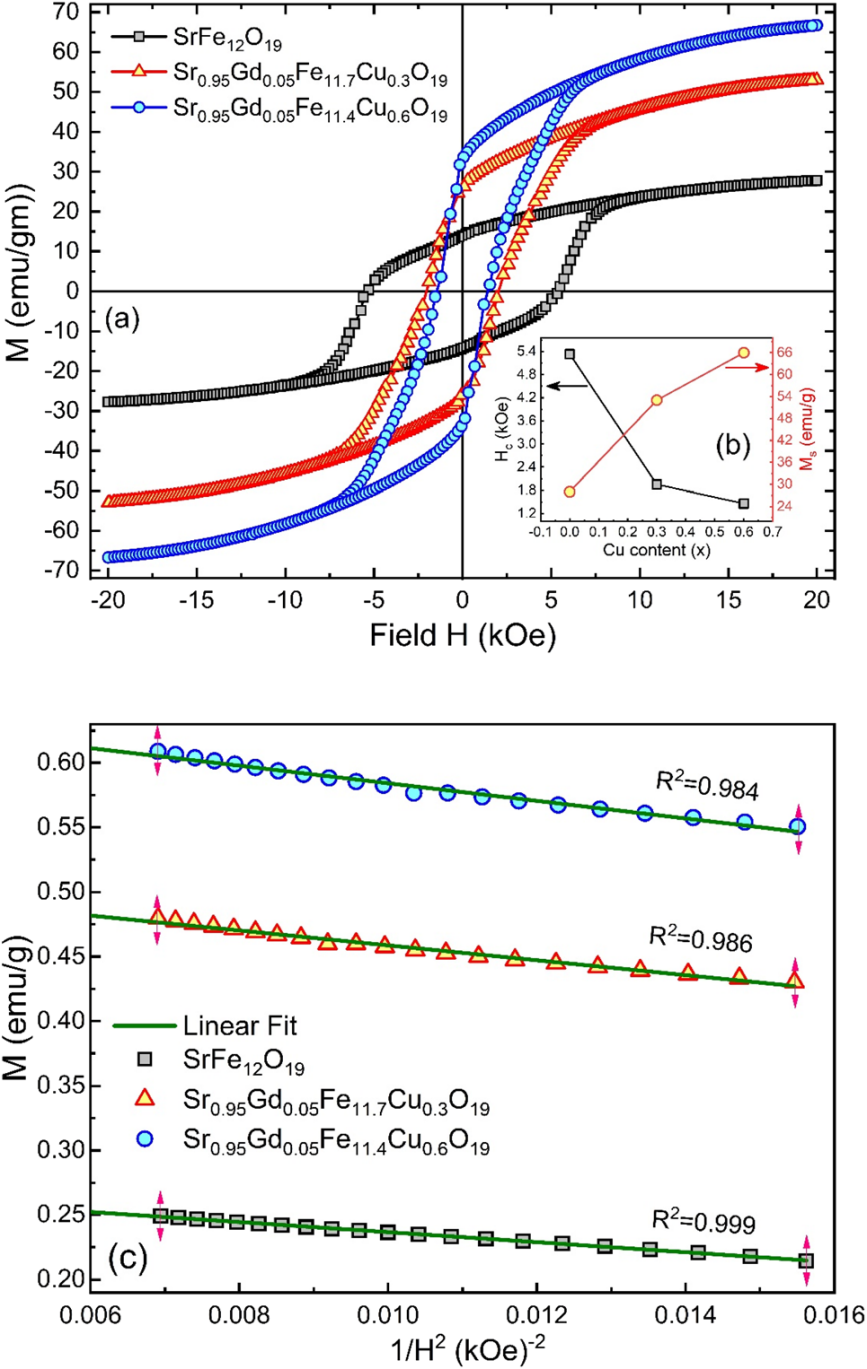
(a) *M*–*H* loops for SrFe_12_O_19_, Sr_0.95_Gd_0.05_Fe_11.4_Cu_0.6_O_19_, and Sr_0.95_Gd_0.05_Fe_11.7_Cu_0.3_O_19_ samples; (b) the inset shows the variations of *M*_s_ and *H*_c_ with doping Cu content in the parent composition. (c) Linear fitting of the variation of M *versus* 1/*H*^2^ curve.

The *M*–*H* loop of pure SrFe_12_O_19_ and Sr_0.95_ Gd_0.05_ Fe_12−*x*_Cu_*x*_O_19_ (*x* = 0.30 and 0.60) nanoparticles are displayed in Fig. 8(a) and the shape of the loops represents the ferromagnetic behaviour of all studied samples. Fig. 8(c) displays the linear fitting of *M versus* 1/*H*^2^ in the higher region of *H* and the data follows the law of approach to saturation (LAS).^50^ The maximum levels of magnetization (*M*_S_) of all samples were determined from the *y*-intercept of the extrapolated line in Fig. 8(c). The variation of *M*_s_ and coercivity (*H*_c_) with Cu concentration has been depicted by the inset Fig. 8(a). Here, *H*_c_ is inversely proportional to *M*_S_, which ensured the magnetic softening of the SFO due to Gd^3+^ and Cu^2+^ substitution and the values of *M*_s_ reached a maximum of 65.2 emu g^−1^, which is suitable for industrial application. The stability of the remanent state of magnetization is described by *H*_c_, which is a specific incoherent mode caused by the rotation of spontaneous magnetization. Table 4 depicts the values of *M*_S_ and *H*_c_. The values of *H*_c_ for SGFCO-1 and SGFCO-2 samples are, respectively, 1.9 kOe and 1.5 kOe, which are much lower as compared to the SFO sample (5.3 kOe). This indicates the decrease in magnetic anisotropy due to the substitution of Gd^3+^ at Sr^2+^ and Cu^2+^ at Fe^3+^.^51,52^ However, the net magnetization (*n*_B_) was determined from *M*_s_ and the molecular mass (*M*) of the studied samples according to the following equation:^53^10
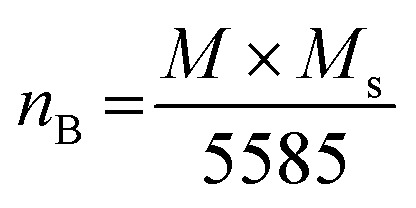


**Table 4:** The effects of Cu^2+^ substitution on the magnetic properties showing maximum magnetization (*M*_s_), coercivity (*H*_c_), remanence (*M*_r_), magnetic moment (*n*_B_), squareness ratio (*S*_r_) and calculated maximum product, (*BH*)_max_ for SFO, SGFCO-1, and SGFCO-2 samples

Sample	*M*_s_ (emu g^−1^)	*H*_c_ (kOe)	*M*_r_ (emu g^−1^)	*M*_r_/*M*_s_	*n*_B_	*S*_r_	(*BH*)_max_ (MGOe)
SFO	27.6	5.3	14.1	0.511	5	0.51	0.24
SGFCO-1	51.6	1.9	25.8	0.500	10	0.5	0.84
SGFCO-2	65.2	1.5	33.2	0.509	12	0.51	1.33

Table 4 displays the values of *n*_B_ and an increased net magnetization was achieved due to the substitution of Gd^3+^ at Sr^2+^ and Cu^2+^ at Fe^3+^ in the SFO sample. Here, the partial substitution of Gd^3+^ at Sr^2+^ led to an increase in the net magnetization as the magnetic moments of Gd^3+^ (8 μB) and Fe^2+^ (4.9 μB) at tetrahedral sites are greater than that of Fe^3+^ (5.9 μB).^54^ On the other hand, as the magnetic moment of Cu^2+^ (1.73 μB) is lower than that of Fe^3+^ (5 μB), Cu^2+^ substitution at Fe^3+^ should lead to the lowering of the net magnetization of SGFCO-1 and SGFCO-2 samples. However, these samples showed higher magnetization than the parent sample (SFO), which is attributed to the site preference of Cu^2+^; as suggested in the literature,^55^ Cu^2+^ preferably occupies an octahedral site. In the M-type hexaferrite, the magnetic moments of Fe are located at the three octahedral (2a, 12k, and 4f2) sites that are parallel to each other, and these moments are coupled in an antiparallel manner to the magnetic moments of Fe located at the tetrahedral (4f1) and trigonal bipyramidal (2b) sites. The magnetic moments within the 4f1 and 2b sites are also parallel to each other. Therefore, the net magnetization arises due to the difference between the magnetization of the octahedral sites (2a, 12k, and 4f2) and the net magnetization of both the tetrahedral and trigonal bipyramidal sites (4f1 and 2b). Since Cu^2+^ prefers to occupy the octahedral site, replacing the Fe^3+^, then the net magnetization of the octahedral sites decreases. From a literature review by P. N. Anantharamaiah *et al.*, it was observed that Cu^2+^ replaces the Fe^3+^ of the 4f2 site with an equivalent amount.^56^ Therefore, the substitution of Cu^2+^ takes part in increasing the net magnetization (*n*_B_) in SGFCO-1 and SGFCO-2. Besides, the squareness ratio (*S*_r_ = *M*_r_/*M*_S_) determines the uniaxial anisotropy contribution in RE-doped nanoparticles generated by the internal strains.^57,58^ The values of *S*_r_ are less than 1 for the studied samples and indicate the presence of an isolated ferromagnetic single domain ^59^. The squareness ratio *M*_r_/*M*_s_ determines the domain state. It can be used to distinguish between single domain (SD), multidomain (MD), and pseudo-single domains (PSD). Indeed, the material can be considered as MD for *M*_r_/*M*_s_ < 0.1, where the magnetization change can be achieved by the domain wall movement in relatively low fields, contrarily to SD (*M*_r_/*M*_s_ > 0.5) where the changes in the magnetization can be realized by its rotation.^60^ Besides, the material can be considered as PSD if *M*_r_/*M*_s_ is between 0.1 and 0.5.^61^

Consequently, the synthesized SGFCO-1 sample falls into PSD as *M*_r_/*M*_s_ = 0.5, while the other two samples fall into the SD as *M*_r_/*M*_s_ > 0.5. Moreover, *M*_r_/*M*_s_ is linked to the magnetic anisotropy and super-exchange interaction between tetrahedral (A) and octahedral (B) ions in the spinel lattice, which depends on the type and number of ions at A and B sites. This distribution affects the magnetization and coercivity of A and B sub-lattices.^62^ The variation in the cationic distribution of Fe^2+^ and Fe^3+^ due to the substitution of Gd^3+^ at Sr^2+^ and Cu^2+^ at Fe^3+^ is the main reason for the gradual variation in *M*_r_/*M*_s_ for the synthesized ferrites. However, some Fe^3+^ exits the spinel lattice due to the formation of the impurity phase of α-Fe_2_O_3_, though the cationic distribution is ruled by the foreign atoms of Cu and Gd in SGFCO-1 and SGFCO-2. Therefore, the *M*_r_/*M*_s_ ratio decreases in SGFCO-1 and then increases in SGFCO-2, depending on the amount (%) of the α-Fe_2_O_3_ phase. The maximum energy density product (*BH*)_max_ for the studied samples was calculated from the equation^63^ as follows:11
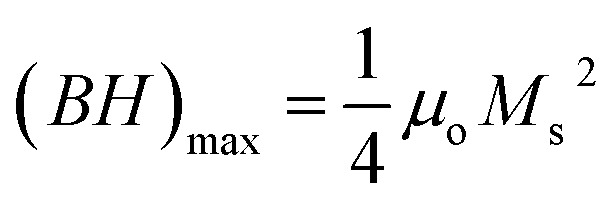
where *μ*_o_ is the permeability constant (*μ*_o_ = 4π × 10^−7^ H m^−1^). The values of (*BH*)_max_ have been included in Table 4 and the maximum value was obtained for the SGFCO-2 sample (1.33 MGOe). It was previously reported that an excess amount of α-Fe_2_O_3_, which remained unreacted, could lead to the weakening of the magnetic properties.^64^ However, in our case, the amount of α-Fe_2_O_3_ decreased due to the substitution of Gd^3+^ and Cu^2+^ in the SFO sample and (*BH*)_max_ increased from 0.24 MGOe to 1.33 MGOe, and *M*_s_ increased from 27.6 to 65.2 emu g^−1^.

### 3.6. Magnetic anisotropy

**Fig. 9:**
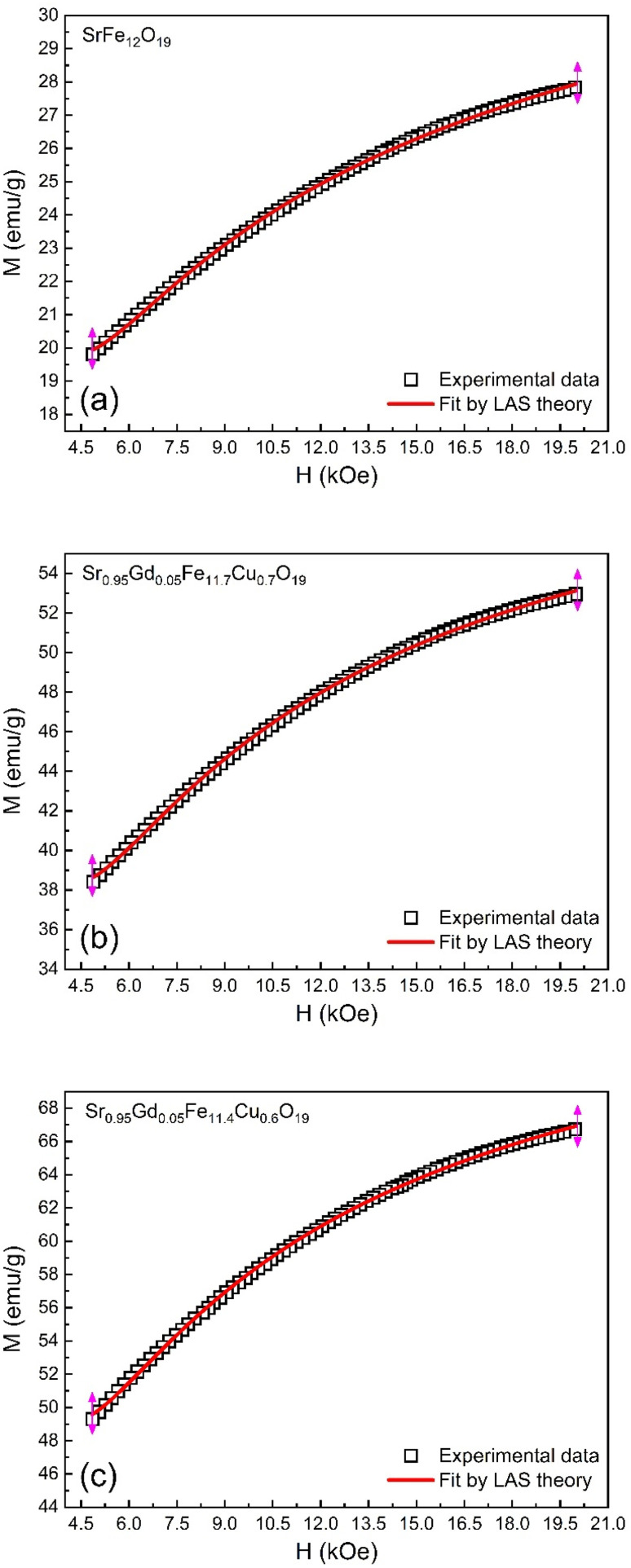
Fitting to the law of approach to saturation (LAS) of magnetization for the compositions of (a) SrFe_12_O_19_, (b) Sr_0.95_Gd_0.05_Fe_11.4_Cu_0.6_O_19_, and (c) Sr_0.95_Gd_0.05_Fe_11.7_Cu_0.3_O_19_.

The magnetic properties of any ferrite samples are dependent on their local crystalline anisotropy. Therefore, the *M*–*H* curves of the studied samples were fitted by the empirical formula of LAS theory, and the equation is expressed as follows:^65^12
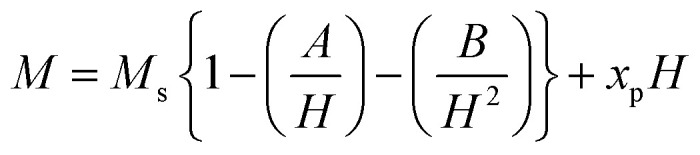
where *A* is the inhomogeneity parameter, *B* is the anisotropy factor and *χ*_p_ defines the high field susceptibility. In addition, *A*/*H* describes the degree of material inhomogeneity while *x*_p_*H* defines the term for forced magnetization caused by the applied field. The terms of *χ* and *A*/*H* vanished for the application of an excessive magnetic field. Another term, 
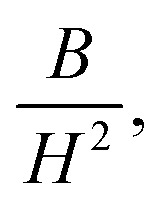
 is connected to the magneto-crystalline anisotropy parameter. Therefore, the *M*–*H* data of Fig. 8(a) has been fitted by eqn (12) for a specific region of *H* (6–20 kOe) and the fittings are depicted in Fig. 9. The values of the statistical coefficient (*R*^2^) confirmed the fitting quality with a high degree of stability. The measured values of *A*, *B* and *χ*_p_ along with *R*^2^ have been included in Table 5. Here, the higher values of the inhomogeneity parameter (*A*) are attributed to the presence of structural defects due to the presence of any secondary phase.^66^ In our present samples, α-Fe_2_O_3_ is the secondary phase as predicted from XRD and FTIR spectra and this phase creates nonmagnetic ion inclusions, as well as structural defects. Moreover, the anisotropy factor, *B*, can be determined from the following equation:13
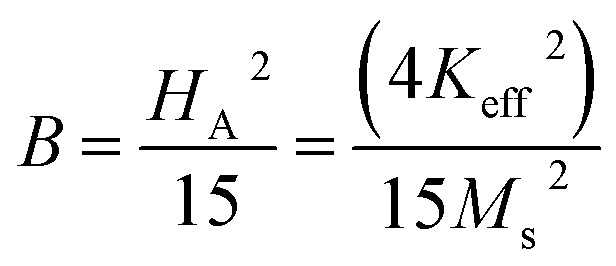
where, *K*_eff_ is the magneto-crystalline anisotropy constant, and *H*_A_ is the anisotropy field. After simplifying eqn (13), the value of *H*_A_ and *K*_eff_ can be determined from the following equations:14
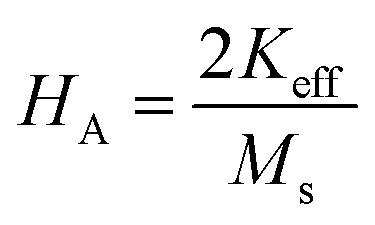
15
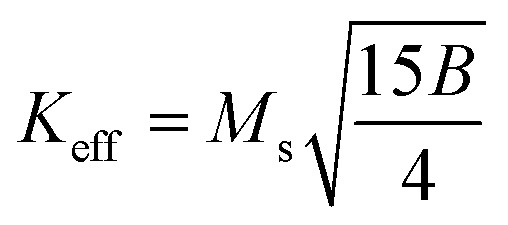


**Table 5:** Values of anisotropy factors (*A* and *B*), magnetic saturation from LAS fitting (*M*_s1_) high field susceptibility (*χ*_p_), anisotropy field factor (*H*_A_), and magneto-crystalline anisotropy (*K*_eff_) along with goodness of the curve fit (*R*^2^) calculated from the fitting of *M*–*H* data (Fig. 6) for the M-type compositions SrFe_12_O_19_ and Sr_0.95_Gd_0.05_Fe_12−*x*_Cu_*x*_O_19_ where *x* = 0.3 and 0.6

Sample	*A* (×10^3^)	*B* (×10^6^)	*χ*_p_ (×10^−5^)	*M*_s1_	*H*_A_ (kOe)	*K*_eff_ (erg cc^−1^)	*R* ^2^
SFO	3.2 ± 0.1	7.2 ± 0.1	8.4	30.7 ± 0.3	10.4	0.7 × 10^4^	0.999
SGFCO-1	3.2 ± 0.1	7.2 ± 0.2	5.7	60.8 ± 0.6	10.4	1.3 × 10^4^	0.999
SGFCO-2	3.1 ± 0.1	6.6 ± 0.2	5.7	76.2 ± 0.7	9.9	1.6 × 10^4^	0.998

**Fig. 10:**
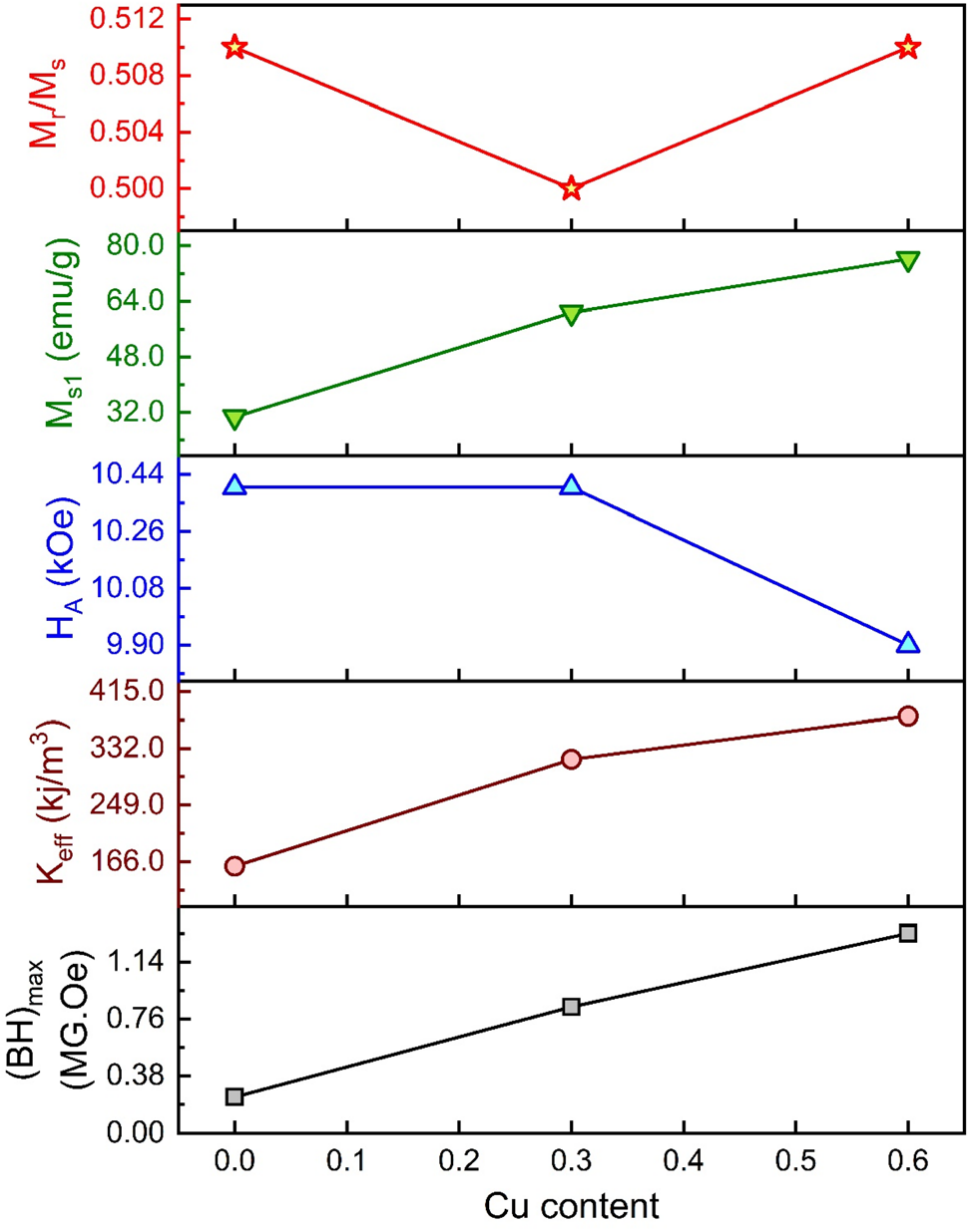
The overall variation of magnetic parameters with Cu content for the M-type compositions of SrFe_12_O_19_, Sr_0.95_Gd_0.05_Fe_11.4_Cu_0.6_O_19_, and Sr_0.95_Gd_0.05_Fe_11.7_Cu_0.3_O_19_.

The values of *H*_A_ and *K*_eff_ are included in Table 5. Here, the deduced *H*_A_ showed fewer variations for substituted Gd^3+^ and Cu^2+^. The overall variation in the magnetic parameters is depicted in Fig. 10.

### 3.7. Temperature-dependent magnetic properties

**Fig. 11:**
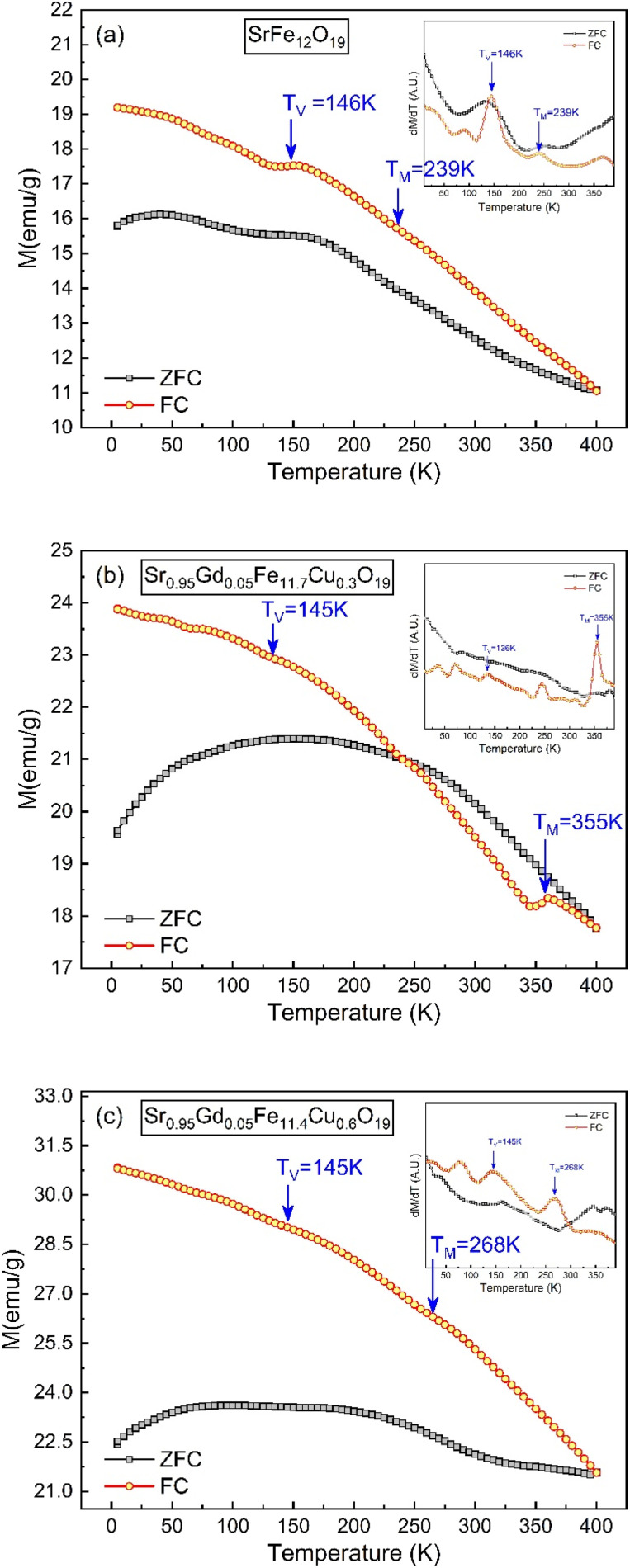
Temperature-dependent magnetization showing variations of ZFC and FC along with the 1st derivative of the ZFC and FC (inset) obtained for (a) SrFe_12_O_19_, (b) Sr_0.95_Gd_0.05_Fe_11.4_Cu_0.6_O_19_, and (c) Sr_0.95_Gd_0.05_Fe_11.7_Cu_0.3_O_19_.

The thermo-magnetization (*M*–*T*) ranging from 10 K to 400 K for SFO, SGFCO-1 and SGFCO-2 is represented in Fig. 11. Here, the measurements were performed under the application of a 100 Oe applied field and the magnetic properties were in the field cooled cooling (FCC) mode between 400 K to 5 K. In addition, the *M*–*T* measurement in the zero-field cooling (ZFC) mode was also measured for the studied samples in the same temperature range. From these *M*–*T* curves, the magnetic moment (emu g^−1^) was higher for the SGFCO-1 and SGFCO-2 samples in the whole temperature run, which is concomitant with the magnetic hysteresis for the 100 Oe applied field. In addition, the magnetization during FCC measurement was increased by lowering the temperature for all studied samples except for a slight saltation for SFO and SGFCO-1 samples. The same type of behaviour was observed for the SrFe_12_O_19_ samples by Gang Qiang^67^ for the 50 Oe applied field. The primary distinction between ZFC and FCC is whether an external magnetic field is dominating throughout the cooling process. In addition, both methods together offer to explain the magnetic interactions in the SFO hexaferrite and different transitions can be identified in the thermal evolution of magnetization. Therefore, transition temperatures have been tracked from the first derivative of magnetization (d*M*/d*T*) of ZFC and FCC data. The variation of (d*M*/d*T*) with temperature (*T*) is illustrated by the inset in Fig. 11(a–c); a jump was observed at ∼145 K in all samples. This peak approximates the temperature of the Verwey transition (*T*_V_ ∼ 120 K) of Fe_3_O_4_, which is a first-order magnetic phase transition related to the change in the magneto-crystalline anisotropy and the ordering of Fe^3+^ and Fe^2+^ ions at the octahedral sites^68^ of the cubic spinel structure. Above this temperature, another jump in d*M*/d*T* was observed in all samples as illustrated in the inset of Fig. 11(a–c). Due to the presence of α-Fe_2_O_3_, a magnetic transition (weak ferromagnetic to antiferromagnetic) may occur at ∼260 K. This transition temperature is known as the Morin temperature (*T*_M_)^68^ and it varies with particle shape, size, and crystallinity. The values of *T*_M_ were observed near 239 K, 355 K and 268 K for SFO, SGFCO-1 and SGFCO-2, respectively. Apart from this, the rare earth moments of Gd are responsible for the higher magnetic potential energy. As the temperature drops to a certain level of separation, the potential energy of the metastable state takes place with an orientation that switches the Gd moments in the opposite direction. Furthermore, the interaction between the adjacent Fe ions builds up a metastable state at a certain level of temperature and the moments of the Fe^3+^ ions changed direction for a short time. Therefore, the huge magnetic potential energy might be released around the transition temperature in the SGFCO-1 sample, resulting in a decrease in the magnetization. However, with the increase in Cu^2+^ content for the FCC mode, the jumping behaviour nearly disappeared as seen in Fig. 11(c) for the SGFCO-2 sample. In principle, the measurement of FCC is dominated by both temperature and external magnetic field, while in the ZFC mode, only the magnetic potential energy develops as the temperature is lowered. Thus, the creation and annihilation of the metastable state in ZFC is solely dominated by the temperature; in contrast in the FCC mode, the diminishing of the metastable state is due to the external magnetic field responsible for the disappearance of jumping behaviour. Therefore, the ZFC/FC tests for the M-type hexaferrite systems revealed interesting behaviour due to the co-substitution of Gd and Cu and detected magnetic transition nature.

## 4. Conclusion

The Cu–Gd-substituted M-type Sr hexaferrites with the formula Sr_0.95_ Gd_0.05_ Fe_12−*x*_Cu_*x*_O_19_ (*x* = 0.30 and 0.60), were successfully prepared *via* the sol–gel method, and calcined at 750 °C in air for 4 hours. However, the substitution of Gd^3+^ and Cu^2+^ ions in SrFe_12_O_19_ increased the unit cell volume and can eliminate the common impurity phase of α-Fe_2_O_3_. The grain sizes were also increased in the co-doped samples and varied from 20 nm to 100 nm holding the nano-plate shape. The saturation magnetization (*M*_s_) increased with the introduction of Gd^3+^ and Cu^2+^ in SrFe_12_O_19_ and *M*_s_ was highest for Sr_0.95_Gd_0.05_Fe_11.4_Cu_0.6_O_19_ (65.2 emu g^−1^) with the lowest coercivity (*H*_c_) of 1.5 kOe as compared to the other two samples. Moreover, the increased number of magneto-crystalline anisotropic factors enabled this composition, resulting in a maximum energy density product, (*BH*)_max_, of 1.33 MGOe. The Sr_0.95_Gd_0.05_Fe_11.4_Cu_0.6_O_19_ composition accumulated a huge magnetic potential energy and suppressed the magnetic transition. The overall properties of Sr_0.95_Gd_0.05_Fe_11.4_Cu_0.6_O_19_ make it a strong contender for use in microwave-absorbing materials and high-density magnetic recording materials, multiple state logic, non-volatile memory and magnetoelectric sensors.

## Conflicts of interest

The authors have no conflict of interest to declare.

## Supplementary Material
